# Removal of Ibuprofen in Water by Bioaugmentation with *Labrys neptuniae* CSW11 Isolated from Sewage Sludge—Assessment of Biodegradation Pathway Based on Metabolite Formation and Genomic Analysis

**DOI:** 10.3390/jox15010005

**Published:** 2024-12-31

**Authors:** Inés Aguilar-Romero, Fernando Madrid, Jaime Villaverde, Esteban Alonso, Juan Luis Santos, Esmeralda Morillo

**Affiliations:** 1 Institute of Natural Resources and Agrobiology of Seville, Spanish National Research Council (IRNAS-CSIC), 41012 Seville, Spain; ines.aguilar@csic.es (I.A.-R.); fmadrid@irnase.csic.es (F.M.); jvillaverde@irnase.csic.es (J.V.); 2 Departamento de Química Analítica, Escuela Politécnica Superior, Universidad de Sevilla, C/Virgen de África, 7, 41011 Seville, Spain; ealonso@us.es (E.A.); jlsantos@us.es (J.L.S.)

**Keywords:** ibuprofen, wastewater, bacterial remediation, metabolites, toxicity, *Labrys neptuniae*

## Abstract

Ibuprofen (IBP) is one of the most consumed drugs in the world. It is only partially removed in wastewater treatment plants (WWTPs), being present in effluent wastewater and sewage sludge, causing the widespread introduction of IBP as an emergent xenobiotic in different environmental compartments. This study describes the use of *Labrys neptuniae* CSW11, recently described as an IBP degrader, through bioaugmentation processes for the removal of IBP from water under different conditions (additional carbon sources, various concentrations of glucose and IBP). *L. neptuniae* CSW11 showed very good results in a wide range of IBP concentrations, with 100% removal in only 4 days for 1 and 5 mg L^−1^ IBP and 7 days for 10 mg L^−1^, and up to 48.4% removal in 28 days for IBP 100 mg L^−1^ when using glucose 3 g L^−1^ as an additional carbon source. Three IBP metabolites were identified during the biotransformation process: 1-hydroxyibuprofen (1-OH-IBP), 2-hydroxyibuprofen (2-OH-IBP), and carboxyibuprofen (CBX-IBP), whose concentrations declined drastically in the presence of glucose. IBP metabolites maintained a certain degree of toxicity in solution, even when IBP was completely removed. The results indicate that *L. neptuniae* CSW11 can be quite effective in degrading IBP in water, but the bioaugmentation method should be improved using CSW11 in consortia with other bacterial strains able to degrade the toxic metabolites produced. A genome-based analysis of *L. neptuniae* CSW11 revealed different enzymes that could be involved in IBP biodegradation, and a potential metabolic pathway was proposed based on the metabolites observed and genome analysis.

## 1. Introduction

Pharmaceuticals are more and more frequently used in veterinary and human medicine worldwide, being those that do not require a medical prescription the most consumed, such as nonsteroidal anti-inflammatory drugs (NSAIDs). After their use, they reach WWTPs, where pharmaceuticals are only partially degraded. Due to the increasing discharge of effluents from WWTPs to the surroundings and the use of the sewage sludge produced as fertilizer in agricultural soils, their presence in a wide variety of environmental matrices has been detected [1,2], being considered emerging pollutants due to their ecotoxicological effects in the ecosystems [3,4].

IBP is one of the most consumed drugs in the world. It is excreted by humans as an unchanged molecule (up to 15%, due to its low solubility and, therefore, bioavailability) and as its metabolites, which can be released into water bodies in higher concentrations than the parent compound [5]. This anthropogenic activity is the cause of its presence in the environment, which is aggravated due to its bioactive nature. The presence of IBP in effluent wastewater and sewage sludge has caused the widespread introduction of IBP in many different environments, such as rivers, lakes, groundwater [6], sea water [7], river sediments [8], and soils [9]. This leads to a pseudo-persistence of IBP in the environment, which may negatively affect humans, animals, microbes, and plants [10]. Therefore, methods for the removal of IBP and other pharmaceutical compounds from different matrices are needed.

The use of specific degrader strains and microbial consortia isolated from matrices with long-term exposure to certain organic contaminants as a method to degrade them has received increased attention from researchers since it is an effective and low-cost methodology and an environmentally friendly technique [11]. IBP’s chemical structure makes it refractive to degradation by microorganisms due to an aromatic ring with branched substitutions in the para position. However, it has been demonstrated that the long-term exposure of activated sludge or sewage sludge (matrices where IBP is accumulated in WWTPs) to IBP results in the generation of autochthonous microorganisms capable of degrading it in these kinds of matrices since they are adapted to such environmental conditions. Microbial bioremediation utilizes the natural metabolic activities of microorganisms to convert harmful pollutants into simpler, less toxic, or non-toxic substances. This method is considered promising due to its durability, cost efficiency, and environmentally friendly nature. However, bioremediation often targets only the original pollutants, even though this can lead to the creation of more dangerous by-products [12,13].

IBP degradation by microorganisms has been studied at the laboratory scale using microbial communities, such as those which used directly activated sludge [14,15], and, to a lesser extent, with bacterial strains, most of them isolated from activated sludge. Only a few bacterial strains able to degrade IBP have been isolated, and most of them have been identified in the last two decades. Bacterial strains belonging to different families have been shown to degrade IBP, such as *Bacillus thuringiensis* B1(2015b) [16], *Serratia marcescens* BL1 [17], *Microccocus yunnanensis* [18], and *Bacillus siamensis* DSI-1 [19]. However, there are only a few isolated bacterial strains capable of mineralizing IBP, most of them belonging to the family *Sphingomonadaceae* (order *Sphingomonadales, class Alphaproteobacteria*): *Sphingomonas* sp. Ibu-2 [20,21], *Sphingobium yanoikuyae* [22], *Rhizorhabdus wittichii* MPO218 [23], or *Sphingopyxis granuli* RW412 [24]. Wittich et al. [25] described the only bacterial consortium constituted by bacteria that do not belong to the *Sphingomonadaceae* family but was able to mineralize IBP, *Pseudomonas citronellolis* RW422, RW423, RW424 (*Pseudomonadaceae*, order Pseudomonadales, class Gammaproteobacteria), although the authors did not provide information about the percentage of IBP concentration mineralized. The degradation of IBP by bacteria with this drug as the only substrate has shown, in most cases, low efficiency, and co-metabolic conditions have often been used, since the presence of structural substrates similar to that of the contaminant to be treated induces the growth of the bacterial population and/or the generation of enzymes required for cometabolic transformation [26].

All these bioremediation studies show very promising results, but they have been conducted at a laboratory level and have not yet been applied in WWTPs or other environmental matrices. The scaling of these technologies is very complex in the environment since microorganisms require special conditions that are not frequently reached (optimal pH and temperature, additional carbon sources, an adequate contaminant level to induce appropriate enzymes for its biodegradation, etc.). However, such appropriate conditions can be more easily controlled in WWTPs, where most of IBP and the rest of pharmaceuticals are received, being the primary source of their release into the environment, since most of them are not completely eliminated after the different treatments applied. Pharmaceuticals remain partially adsorbed on sewage sludge used as fertilizers in agricultural soils, and other fractions dissolved in the effluent waters generated in the WWTP.

Therefore, it is imperative to continue the research about IBP degraders and bioremediation technologies that can be implemented in the future to eliminate this drug, especially at the WWTP level. To fulfill the above-mentioned research gaps, this study aimed to investigate in depth the pure bacterial strain *Labrys neptuniae* CSW11 isolated in a previous study from sewage sludge in a WWTP [27], which presented the ability to remove IBP from a solution. The effectivity of CSW11 in a wide range of concentrations in solution was also studied, as well as in the presence of different carbon sources. The kinetics of IBP biotransformation by CSW11 were investigated, both supplying IBP as a sole carbon source and with increasing concentrations of glucose supplementation. The identification of metabolites formed during the process was carried out and their potential toxicity throughout the bioremediation process was assessed. The possible degradation pathway of IBP by *L. neptuniae* CSW11 was investigated. CSW11 was characterized at a genomic level and the genomic sequences were compared to the genes of other IBP-degrading bacteria obtained from the literature and databases in order to propose a potential IBP degradation pathway.

## 2. Materials and Methods

### 2.1. Chemicals and Bacterial Strain

Analytical standards of IBP (purity > 98%) and IBP sodium salt (purity > 98%) were purchased from Sigma-Aldrich (Madrid, Spain). *Labrys neptuniae CSW11* was isolated from an IBP-degrading consortium (C7) obtained through an enrichment culture with IBP as the sole carbon source of fresh sewage sludge collected from a WWTP in Seville (Southwest Spain) described previously in the work of Vargas-Ordóñez et al. [13]. CSW11 was isolated by Aguilar-Romero et al. [27] from a C7 consortium plated on both MSM-agar medium and LB-agar medium in the presence of IBP (200 mg L^−1^), and was identified by a comparison of its 16S rRNA sequence with the NCBI database using the BLAST sequence similarity search program, and deposited in the GenBank database with the accession number OQ859978. All solvents used were HPLC-grade. The mineral salt medium (MSM) and Luria-Bertani (LB) broth were prepared with analytical-grade chemicals (Sigma-Aldrich, Madrid, Spain) with the composition described in the work of Aguilar-Romero et al. [27], and were autoclaved at 121 °C for 20 min before using. Phylogenetic tree analyses based on the 16S rRNA sequences were performed using the maximum-likelihood and neighbor-joining algorithms with 1000 bootstrap replications in MEGA version 11 software [28] to show the evolutionary distances between *L. neptuniae* CSW11 and the closest species (based on the best BLAST matches).

### 2.2. Inoculum Preparation

CSW11 was grown in LB medium in the presence of IBP sodium salt (200 mg L^−1^) (30 ± 1 °C, 150 rpm) and collected at the beginning of the stationary phase through centrifugation at 7000 rpm (10 min). The pellet obtained was washed with MSM solution to completely remove the IBP previously added and suspended in MSM solution. Bacterial growth was monitored by optical density (OD_600nm_) (UV-3100 spectrophotometer, VWR, Radnor, PA, USA) and by colony-forming units (CFUs) of serial dilutions on LB-agar medium plates.

### 2.3. Biodegradation Assays in Solution with Labrys Neptuniae CSW11

CSW11 was inoculated in triplicate at an initial cell density of 10^8^ CFU mL^−1^ in flasks with 50 mL of MSM medium spiked with IBP (10 mg L^−1^). Microcosms were incubated in triplicate under aerobic conditions on a temperature-controlled rotary shaker at 30 °C and 180 rpm for 28 days. Firstly, CSW11 was inoculated in the presence of diverse external carbon sources to determine the possibility of cometabolic degradation. For this purpose, biodegradation experiments were performed under the same conditions, and glucose (1 g L^−1^), sodium acetate (1 g L^−1^) or yeast extract (1 g L^−1^) were added to the MSM medium; according to the results obtained, glucose was selected to carry out the kinetic studies. To observe any IBP abiotic removal, non-inoculated sterile controls were also prepared. At periodic intervals, 1 mL of culture medium from each flask was removed and frozen and thawed three times to break cell walls and recover IBP accumulated in the microbial biomass. Thereafter, the samples were centrifuged at 11,000 rpm for 2 min, and IBP concentration determined in the supernatant as explained in Section 2.4.

The capacity of *L. neptuniae* CSW11 for IBP biotransformation was assayed using a wide IBP concentration range. In order to select an appropriate range, the inhibitory concentration of IBP required to reduce *L. neptuniae* CSW11 bacterial growth (half maximal inhibitory concentration, IC_50_) was firstly studied. For this purpose, the bacterial isolate was cultivated in triplicate in LB medium supplemented with different concentrations of IBP sodium salt (10, 50, 100, 500 and 3000 mg L^−1^). Similar cultures without IBP were run in parallel as a reference of normal bacterial growth. All cultures were incubated under aerobic conditions at 30 °C on a shaker at 180 rpm. Bacterial growth was analyzed in all cultures after 24 h by measuring the OD_600nm_ to obtain the percentage of bacterial viability (OD_600nm_ measured in each culture with a certain IBP concentration divided by OD_600nm_ in the cultures without IBP, multiplied by 100). The IC_50_ value was estimated as a linear function of the logarithm of IBP concentration (mg L^−1^) and of bacterial viability (%) using the Microsoft Excel program.

Based on the IC_50_ value obtained, low (1 and 5 mg L^−1^) and high (50 and 100 mg L^−1^) initial IBP concentrations were selected, and *L. neptuniae* CSW11’s biotransformation capacity was assayed in triplicated in MSM medium in the absence of glucose and with two different glucose concentrations (1 and 3 g L^−1^) as an additional carbon source. The cultures were inoculated with the bacterial strain at 10^8^ CFU mL^−1^ and incubated in the same previous conditions.

### 2.4. Determination of Ibuprofen and Its Metabolites

IBP concentration was determined in the supernatant solutions using an HPLC analyzer with a UV–vis detector (LC-2010AHT, Shimadzu, Kyoto, Japan), a C-18 column (4 × 150 mm), mobile phase methanol/water (80:20) at pH 3 with orthophosphoric acid (1%), flow rate of 1.2 mL min^−1^, detection wavelength 210 nm and injection volume 25 µL. The IBP detection limit was 0.05 mg L^−1^.

The analysis of IBP metabolites was performed by high-performance liquid chromatography with tandem mass spectrometry. Chromatographic analyses were carried out on an Agilent 1290 infinity II series HPLC (Agilent, Santa Clara, CA, USA) equipped with a vacuum degasser, a binary pump, an autosampler and a thermostated column compartment. The chromatographic column was a Halo C-18 (50 mm × 4.6 mm i.d., 2.7 µm particle size) (Teknokroma, Spain). The column was protected by a Halo C18 (5 mm × 4.6 mm i.d., 2.7 µm particle size) guard column (Teknokroma, Barcelona, Spain). The mobile phase was composed of water (0.1% of formic acid) (solvent A), and acetonitrile (0.1% of formic acid) (solvent B) operating by gradient elution. The gradient program was as follows: 0–2 min, isocratic 10% solvent B, linear gradient from 10% to 30% of solvent B in 2 min, linear gradient from 30% to 100% of solvent B in 4 min, and held for 7 min. Finally, it was back to initial conditions in 5 min. The flow rate was fixed at 0.6 mL min^−1^. The injection volume was 10 µL and the column temperature was maintained at 35 °C. MS measurements were carried out with a 6495 QqQ-MS (Triple Quadrupole Mass Spectrometer, Radnor, PA, USA) instrument equipped with an electrospray ionization source (Agilent). The ionization of analytes was conducted using the following settings: MS capillary voltage 4000 V, drying-gas flow rate 11 L min^−1^, drying-gas temperature 350 °C, nebulizer pressure 40 psi and Fragmentor 166 V. Two Multiple Reaction Monitoring (MRM) transitions were selected for quantification and confirmation of target compounds in the analyzed samples. The selected transitions and the intensity ratio (Quantification/Confirmation ions) are shown in Table 1. The mass spectrometer was operated in both positive and negative ESI modes. The optimized collision energy for each MRM transition is also shown in Table 1. The limit of quantification was 1 µg/L in the case of 1-hydroxyibuprofen (1-OH-IBP) and 2-hydroxyibuprofen (2-OH-IBP), and 10 µg/L for carboxyibuprofen (CBX-IBP). The analysis of other possible IBP metabolites was carried out according to Salgado et al. [29]. For the separation of compounds, 20 μL of the sample extract was injected into a Kinetex^®^ Polar C18 analytical column (50 mm × 3.0 mm i.d., 2.6 µm particle size) from Phenomenex, Torrance, CA, USA, protected by a SecurityGuard™ ULTRA C18 guard column (2 mm × 3 mm i.d.) (Phenomenex, Torrance, CA, USA) and maintained at a temperature of 35 °C. The mobile phase was composed of water (0.1% of formic acid) (solvent A) and acetonitrile (0.1% of formic acid) (solvent B) operating by gradient elution. The elution protocol started with 5% solvent B, ramped up to 100% solvent B over 15 min, held at 100% solvent B for 5 min, returned to initial conditions over 3 min, and was held for 2 min for re-equilibration, making the total run time 25 min. Each extract was analyzed in MRM mode, with specific conditions detailed by Salgado et al. [29].

### 2.5. Models of Biotransformation Kinetics

The IBP biotransformation plots were modelized (whenever possible) using the Excel file provided by FOCUS (2006) [30] for degradation kinetics and the Solver tool (Microsoft statistical package), and optimized by using the least-squares method. In general, biotransformation curves fitted well to three first-order kinetic models: a simple first-order model (SFO), a biphasic first-order sequential model (Hockey-Stick, HS), and a first-order multicompartment model (FOMC) using the following equations:
Mt = M_0_ e^−kt^
(SFO)DT_50_ = ln2/K 
(SFO)Mt = M_0_ e^−k1^ tb e^−k2(t − tb)^
(HS)DT_50_ = (ln 100/100−50)/K_1_if DT_50_ ≤ tb(HS)DT_50_ = tb + (ln 100/100−50) − K_1_ tb)/K_2_ if DT_50_ > tb(HS)Mt = M_0_/((t/β) + 1)^α^
(FOMC)DT_50_ = β (2 (1/α) − 1) 
(FOMC)α = 1/(N − 1)
(FOMC)β = (M_0_/1 − N)/[K(N − 1)]
(FOMC)
where Mt and M_0_ are the concentrations of the remaining IBP (mg L^−1^) at time t and immediately after spiking the solution, respectively, and K is the rate constant of degradation (day^−1^). In the HS model, K_1_ and K_2_ are the rate constants of degradation (day^−1^) for the fast and the slow fractions, respectively, and tb is the time at which the rate constant changes. For the FOMC model, α corresponds to the shape parameter determined by the coefficient of variation of K values, and β is the location parameter. DT_50_ refers to the time required for the IBP concentration to reach half of its initial value. The scaled error values and chi-square (χ^2^) (*p* < 0.05) were used to estimate the appropriateness of the models.

### 2.6. Ecotoxicity Bioassays

In the IBP biotransformation processes in solution using *L. neptuniae* CSW11, the inhibitory effect of IBP and/or possible metabolites produced was studied using ecotoxicity assays according to ISO 11348-3 [31] based on the light emission of freeze-dried luminescent bacteria *Aliivibrio fischeri*. After filtering the solutions to remove the remaining particulate matter, they were serially diluted with NaCl. The decrease in luminescence after 15 min contact was measured using a Microtox model 500 Analyser (Modernwater, York, UK, and compared with the control. The EC_50_ value was calculated as the IBP concentration (% *v*/*v*) with a toxic effect on 50% of the bacterial population, and was expressed in Toxic Units (TU): TU = 100/EC_50_ [32].

### 2.7. Total DNA Extraction and Genome Analysis

The genome of the *Labrys neptuniae* strain CSW11 was analyzed to determine the potential genes involved in ibuprofen biodegradation. Total DNA was obtained from a pure culture of *L. neptuniae* CSW11 grown overnight in LB medium spiked with 200 mg L^−1^ of IBP at 30 °C on a rotary shaker at 180 rpm using the G-spin™ Total DNA Extraction Kit (iNtRON Biotechnology, Inc., Seongnam-si, Republic of Korea). DNA quantity and quality were assessed using NanoDrop 2000 (Thermo Fisher Scientific, Agawan, MA, USA) and DNA integrity was checked by 1.5% agarose gel electrophoresis. The DNA sample was submitted to the STAB VIDA laboratory (Caparica, Portugal) to obtain the genome sequence by following a whole-genome shotgun strategy on an Illumina Novaseq platform (2 × 150-bp paired-end reads). The analysis of the generated raw sequence data was performed using CLC Genomics Workbench 22.02 software. The high-quality sequence reads obtained after a trimming analysis were used to perform a de novo genome assembly approach using an algorithm based on de Bruijn graphs [33]. Then, the genome assemblies were evaluated using QUAST 5.0.2. The assembled genome sequence was annotated with Rapid Annotations using the Subsystems Technology (RAST) server version 2.0 [34]. The Whole-Genome Shotgun project of *L. neptuniae* CSW11 was deposited at DDBJ/ENA/GenBank under accession JAVSCS000000000 (BioProject: PRJNA1018852). Genetic annotation was also performed using NCBI Prokaryotic Genome Annotation Pipeline (PGAP) for genome submission to GenBank [35].

## 3. Results and Discussion

### 3.1. IBP Biotransformation in Aqueous Solution by L. neptuniae CSW11 Under Different Conditions

**Figure 1:**
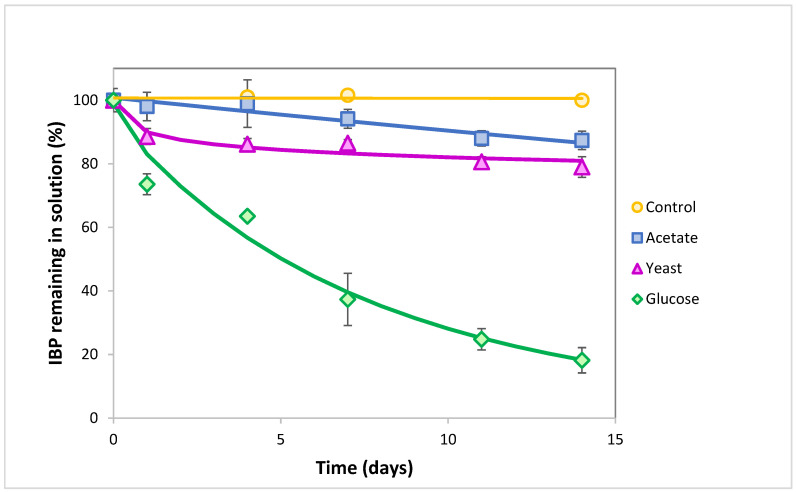
Ibuprofen biotransformation capability by *Labrys neptuniae* CSW11 in the presence of acetate, yeast extract or glucose (1 g L^−1^) as additional carbon sources, in comparison to control experiments without inoculation with CSW11. Error bars indicate standard deviation.

**Figure 2:**
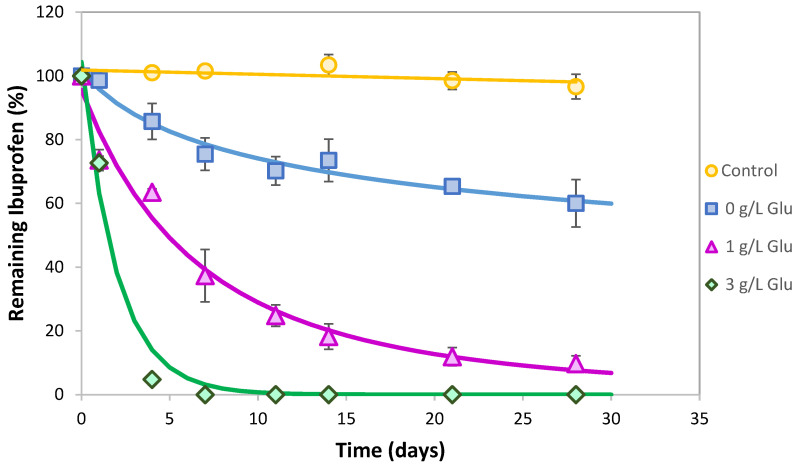
Ibuprofen (10 mg L^−1^) biotransformation capability by *Labrys neptuniae* CSW11 in the absence and in the presence of glucose (1 and 3 g L^−1^) in comparison to control experiments without inoculation with CSW11. Error bars indicate standard deviation.

As *L. neptuniae* had not been described as an IBP degrader until very recently [27], it is interesting to carry out a more in-depth study of its behavior. First, estimations of the inhibitory concentration of IBP for *L. neptuniae* CSW11 growth (concentration of IBP required to reduce the bacterial growth rate by 50%) were carried out and an IC_50_ value of 217 mg L^−1^ was observed (Figure S1 in Supplementary Information). Given the low IBP concentrations found in wastewater and sewage sludge [36], this IC_50_ value indicates that *L. neptuniae* CSW11 is able to grow in such environments, even as a monosubstrate. Different substrates such as glucose, yeast extract or sodium acetate (all of them at 1 g L^−1^) were studied to observe if IBP biotransformation by *L. neptuniae* could be improved. The best results were obtained with glucose (Figure 1), reaching about 80% IBP (10 mg L^−1^) removed in 14 days, while with yeast extract or sodium acetate, IBP removal was ≤20%. In order to observe glucose influence, complete IBP biodegradation curves using *L. neptuniae CSW11* were carried out for 28 days in the absence and in the presence of 1 and 3 g L^−1^ glucose (Figure 2). An increased IBP removal was observed as the glucose concentration increased, decreasing drastically DT_50_ from 64.5 days when no glucose was used, to only 5.2 and 1.4 days when using 1 and 3 g L^−1^ glucose, respectively (Table 2). It is worth noting that 100% IBP degradation was reached in about 7 days with 3 g L^−1^ glucose (Figure 2), indicating a huge effect of glucose on IBP removal. The addition of glucose as an external carbon source assists in the growth of biomass, and such a large inoculum size could overcome the toxicity of both IBP and the metabolites produced, increasing its biotransformation [37], and/or it could produce enzymes required for a cometabolic transformation [26].

**Figure 3:**
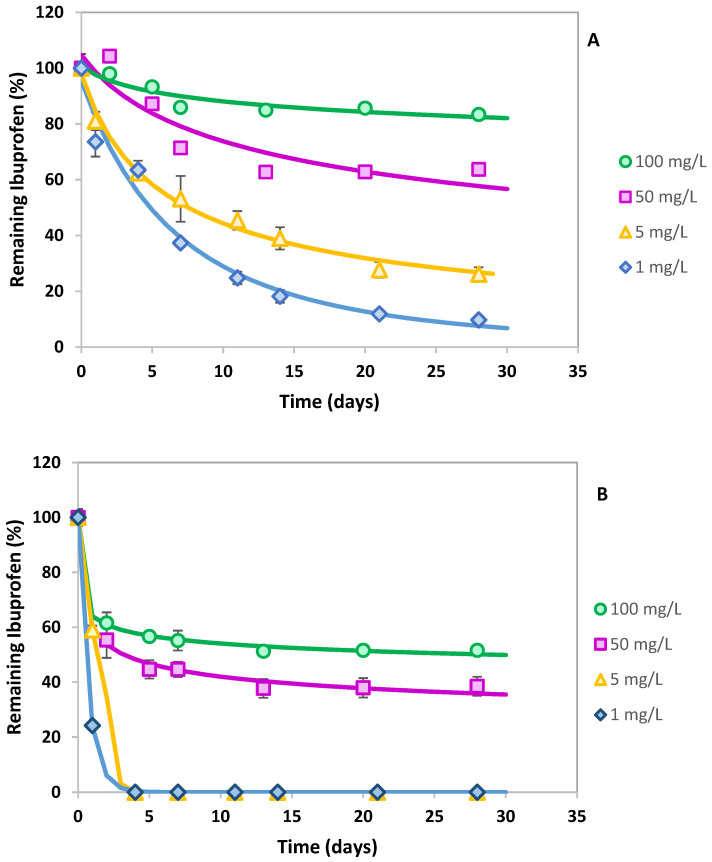
Influence of different ibuprofen concentrations on the removal efficiency of *Labrys neptuniae* CSW11 in the presence of glucose 1g L^−1^ (**A**) and 3g L^−1^ (**B**). Error bars indicate standard deviation.

Figure 3 shows the influence of different IBP concentrations (1–100 mg L^−1^) in its biotransformation by *L. neptuniae* in the presence of glucose 1 and 3 g L^−1^, and the corresponding kinetic parameters are also included in Table 2 (IBP sodium salt was used for concentrations of 50 and 100 mg/L due to the low IBP aqueous solubility, 21 mg L^−1^). Since IC_50_ calculated for *L. neptuniae* CSW11 viability as a function of IBP concentration was 217 mg L^−1^, the concentrations selected were all below this value. In terms of efficiency, all the concentrations tested showed a faster removal rate when using 3 g L^−1^ glucose, decreasing the DT_50_ values drastically and increasing the extent of removal (Table 2). The high difference in kinetic parameters between 1 and 3 g L^−1^ glucose in the highest concentration is especially noteworthy (100 mg L^−1^), reducing the DT_50_ values from more than 30,000 days to only 29 days. And it should also be noted that when using 3 g L^−1^ glucose, 100% IBP removal was reached in only 3–4 days when the two lowest concentrations were used, 1 and 5 mg L^−1^ (Figure 3). This would indicate that even at very low levels of IBP, such as those commonly found in WWTPs, the adequate enzymes for its degradation are induced in the presence of glucose.

The results obtained in this study are similar to those of Marchlewicz et al. [38], who observed the biodegradation of 20 mg L^−1^ IBP in 6 days by *Bacillus thuringiensis* B1(2015b) in the presence of glucose (1 g L^−1^), those of Show et al. [37], with a biodegradation of 15 mg L^−1^ IBP in 5 days of incubation using the strain *Microbacterium paraoxydans* in the presence of yeast (0.3%), and the study of Xu et al. [17], with 30 mg L^−1^ degraded in 5 days by *Serratia marcescens* BL1. But this study’s results are much better than those obtained using the *Patulibacter* sp. strain I11 [39], *Pseudoxanthomonas* sp. DIN-3 [40], *Nocardioides carbamazepini* sp. nov. [41], or *Rhizobium daejeonense* IBU_18 [42]. However, IBP removal is not as high as that obtained with other IBP-degrading strains capable of mineralizing IBP. The strain *Sphingopyxis granuli* RW412 mineralized up to 456.5 mg L^−1^ IBP in 74 h [24], *Sphingomonas* sp. Ibu-2 mineralized up to 500 mg L^−1^ in 80 h [20], and *Variovorax* Ibu-1 mineralized 200 mg L^−1^ in 75 h [43].

Species of the genus *Labrys* have been isolated from different environments: plant rhizosphere, such as *L. okinawensis and L. miyagiensis* [44], and *Labrys soli* sp. [45]; sediments, such as L. *methylaminiphilus* [46] or *L. portucalensis* [47]; water, such as *L. wisconsinensis* [48]; and soil, such as *Labrys* sp.KNU-23 [49]. The species *Labrys neptuniae* was isolated for the first time from root nodules [50], and, as far as we know, *L. neptuniae* is mentioned to assign some DGGE bands in a phylogenetic analysis of the microbial community in activated sludge from a reactor in WWTP only in two works by Yan et al. [51,52], which is quite related with the origin of *L. neptuniae* CSW11. However, *L. neptuniae* has not been previously described as a degrader of either IBP or any other contaminant, and, therefore, it is very important to highlight this novelty. It should also be emphasized that none of the other species belonging to the genus *Labrys* previously mentioned have been described as degraders of contaminants, except *Labrys portucalensis* and, more specifically, *L. portucalensis* F11, which degrade a wide variety of pharmaceutical products [53,54,55,56,57,58] as well as a variety of POPs (Persistent Organic Pollutants) [47,59,60,61,62].

**Figure 4:**
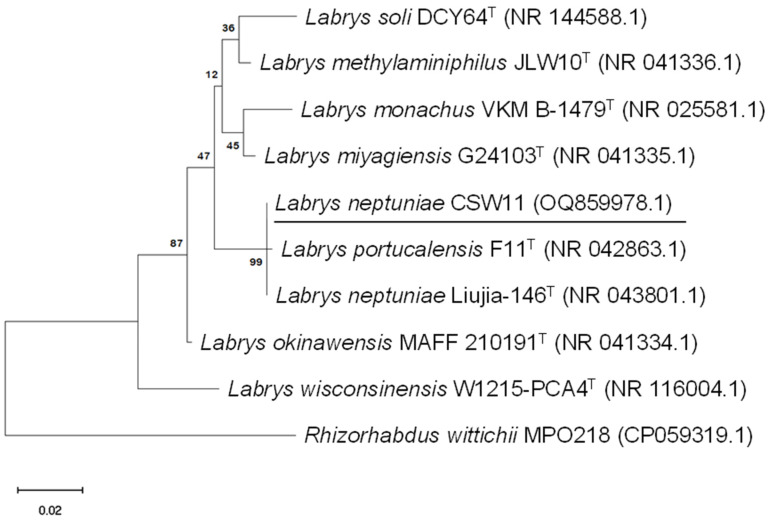
Maximum likelihood tree based on 16S rRNA genes showing the phylogenetic relationships between the strain *L. neptuniae* CSW11 isolated from the ibuprofen enrichment culture and other species of the same genus. *Rhizorhabdus wittichii* MPO218 was used as an outgroup. Bootstrap values shown at the branch nodes are based on 1000 replicates. The scale bar represents 0.02 substitution per nucleotide. GenBank accession numbers are in parentheses.

The phylogenetic affiliation of *L. neptuniae* CSW11 and type strains of species belonging to the genus *Labrys* (indicated with the subscript “T”) is shown in Figure 4. This phylogenetic tree shows the close relationship between the isolate *L. neptuniae* CSW11 and the degrading strain *L. portucalensis* F11, which share most of genes involved in metabolic pathways since they only differ by two nucleotides out of around 1400 base pairs in their 16S rDNA genes.

The similarity values obtained from the phylogenetic analysis were included in a table in a previous study [27], where the similarity percentages for taxonomic assignment at the species level to each isolate are indicated. Taxonomic identification was carried out by comparison with the NCBI database (National Centre for Biotechnology Information, Bethesda, MD, USA) using the BLASTn algorithm. Moreover, the genome of the CSW11 strain was analyzed using the Type Strain Genome Server (TYGS) and the Genome BLAST Distance Phylogeny (GBDP) method, identifying *Labrys neptuniae* LMG 23578 as the closest related species. The ibuprofen-degrading strain *Rhizorhabdus wittichii* MPO218 was used as an outgroup. The *Rhizorhabdus* genus belongs to the order Hyphomicrobiales, as does the *Labrys* genus. In addition, *R. wittichii* was detected in the consortium from which all strains used in this study were isolated. The relative abundance of this species, as well as the total biomass of the consortium, increased dramatically in a culture with ibuprofen as the sole carbon source [27].

### 3.2. Detection of the Main Metabolites in IBP Biotransformation by L. neptuniae CSW11

**Figure 5:**
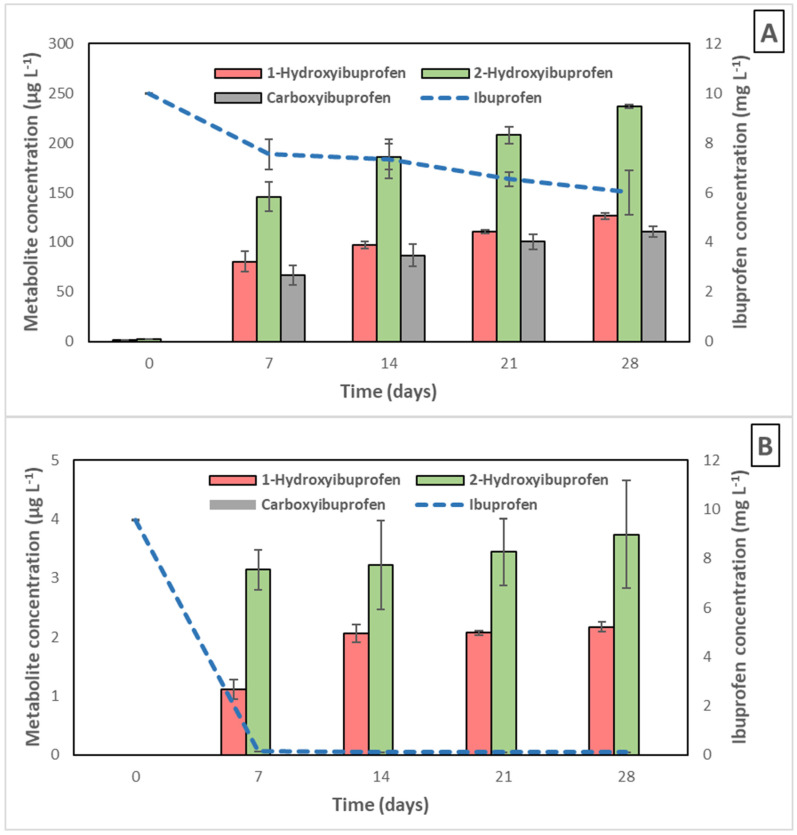
Evolution of ibuprofen (10 mg L^−1^) and metabolites detected during its biotransformation by *Labrys neptuniae* CSW11 in solution in the absence (**A**) and presence of glucose (3 mg L^−1^) (**B**).

In order to observe the production of IBP metabolites throughout its biodegradation process by *L. neptuniae* CSW11, an HPLC with tandem mass spectrometry (MS/MS) technique was used to measure samples corresponding to the IBP (10 mg L^−1^) biotransformation curve in the absence of glucose in comparison to that in the presence of glucose (3 g L^−1^). The results obtained are shown in Figure 5. Three metabolites could be identified: 1-hydroxyibuprofen (1-OH-IBP), 2-hydroxyibuprofen (2-OH-IBP), and carboxyibuprofen (CBX-IBP). In order to detect other possible metabolites, specific conditions detailed by Salgado et al. [29] were also used, but no new metabolites were found. Figure S2 shows the LC-MS/MS chromatograms and mass spectra of IBP and the metabolites detected.

The presence of the metabolites 1-OH-IBP and 2-OH-IBP indicates that hydroxylation has taken place through the side chains of the ring. In the absence of glucose, the concentration of IBP decayed from 10 mg L^−1^ to 6.0 mg L^−1^ after 28 days (40% IBP removed), and the previously mentioned three metabolites were detected with concentrations in the range of 80–126 µg L^−1^ for 1-OH-IBP, 146–237 µg L^−1^ for 2-OH-IBP, and 67–111 µg L^−1^ for CBX-IBP. In contrast, in the presence of glucose 3 mg L^−1^, the IBP concentration decayed from the initial 10 mg L^−1^ to 0.0 mg L^−1^ after only 7 days, and the metabolites were detected at much lower concentrations, in the range of only 1.12–2.17 µg L^−1^ for 1-OH-IBP and 3.14–3.74 µg L^−1^ for 2-OH-IBP, and CBX-IBP was not detected. The absence of CBX-IBP is in agreement with the results observed by Ferrando-Climent et al. [63], who reported that CBX-IBP disappeared faster in activated sludge than the hydroxylated metabolites.

These data indicate the high effectiveness of the use of glucose as a growth substrate together with *L. neptuniae* CSW11 not only for IBP removal from water, but also to avoid the accumulation of IBP metabolites. These metabolites were detected in WWTPs. Ferrando-Climent et al. [63] observed maximum concentrations in influent wastewater samples for IBP, 1-OH-IBP, 2-OH-IBF, and CBX-IBF of 13.7, 5.8, 94.0 and 38.4 μg L^−1^, respectively, which remained in effluent wastewater samples at concentration of 1.9, 1.4, 5.9 and 10.7 μg L^−1^, respectively. Aymerich et al. [64] detected 20 ng/L for CBX-IBP, 1100 ng/L for 1-OH-IBP, and 7800 ng/L for 2-OH-IBP in influent wastewater.

The IBP metabolites detected have been previously observed in biodegradation studies using both microbial consortia and isolated bacterial strains to remove IBP from water. They were more frequently observed when using microbial consortia, and most of the studies were carried out using activated sludge from WWTPs [63,65,66]. These authors suggested that the first reaction in IBP biodegradation is the transformation of its aliphatic chain. Hydroxylation is the primary mechanism for IBP biodegradation.

However, the presence of these detected metabolites is scarcer in those IBP biodegradations carried out using isolated bacterial strains. In some of the five degradation pathways proposed, these metabolites are present [26]. Marchlewicz et al. [16] observed 2-OH-IBP in the degradation process using *Bacillus thuringensis* B1(2015b), but 2-(4-hydroxyphenyl)-propionic acid, 1, 4-hydroquinone and 2-hydroxy-1,4-quinol were also detected as metabolites. They proposed an IBP biodegradation pathway that also used the IBP-degrading bacteria *Citrobacter freundii* PYI-2 and *Citrobacter portucalensis* YPI-2 isolated by Chopra and Kumar [19] since they detected the same metabolic intermediates. Lu et al. [40] used *Pseudosantomonas* sp. DIN-3 to degrade IBP and observed the presence of the metabolites 1-OH-IBP and CBX-IBP that, together with other detected metabolites, made it possible to propose a possible degradation pathway. Salgado et al. [29] carried out metabolite identification of IBP biodegradation by *Patulibacter medicamentovorans,* identifying 22 metabolites. They proposed the hydroxylation and carboxylation of IBP, and 1-OH-IBP and CBX-IBP were detected. Moreover, they found metabolites coming from a sequence of oxidation of methyl groups in the metabolite CBX-IBP molecule, being a detoxification mechanism which produced more soluble metabolites and with lower acute toxicity.

### 3.3. Ecotoxicity Bioassays

Few studies have reported the toxicity of IBP in living organisms, and most of them from aquatic ecosystems, demonstrating that it is harmful to several aquatic species [67]. IBP toxicity towards bacteria has been also reported. In this sense, shifts in associated bacterial community in activated sludge system [68] or in a consortium from sewage sludge after acclimation with IBP [27] have been reported. Di Nica et al. [69] also observed altered bioluminescence in *Allivibrio fischeri* with IC_50_ values in the range of 13.77–15.9 mg L^−1^. Such altered bioluminescence was used in the present study to carry out toxicity measurements using Microtox bioassays throughout the bioremediation process of IBP in solution at the beginning and at the end of biodegradation experiments (28 days) using *Labrys neptuniae* CSW11 in the presence of different IBP and glucose concentrations (Table 3). In spite of the difference in IBP initial concentrations (10, 50 and 100 mg L^−1^) and their respective increased Toxic Units (TUs, 2.25, 3.98, and 5.68), all of them could be classified as belonging to Class III according to the classification of Persoone et al. [70], presenting Acute Toxicity (1 < TU < 10). Aguilar-Romero et al. [27] observed that even an initial IBP concentration of 500 mg L^−1^ also presented an Acute Toxicity level (TU 8.87). Only in the case of initial IBP 10 mg L^−1^ did the toxicity level decrease to Class II (Slight Acute Toxicity) at the end of the experiment (0.4 < TU < 1), which was maintained even when no IBP was present in the system, as in the case of using 3 g L^−1^ glucose. It is indicating that the metabolites produced through the process (previously commented) should maintain this level of toxicity, although that their concentration is quite different in the system without glucose in comparison to that in the presence of glucose, as previously mentioned (Figure 5). In the case of an initial IBP concentration of 50 mg L^−1^, its remaining concentration at the end of the experiments was lower, but within the range of Acute Toxicity. For IBP 100 mg L^−1^ in the presence of glucose 3 g L^−1^, the remaining concentration was 51.6 mg L^−1^, but the system showed High Acute Toxicity (10 < TU < 100), even more toxic than with IBP 100 mg L^−1^, indicating that the metabolites produced through this process (and/or their higher concentrations) are even more toxic than the parent contaminant. The results of the ecotoxicity test indicate that, although IBP was completely removed by *L. neptuniae* CSW11, some of its toxic metabolites could remain in the system, some of them probably not detected by the analytical methods used in this work. This indicates that assessing only the absence of IBP concentrations after applying a bioremediation treatment is not sufficient to confirm a decrease in the toxicity since it could be due to the presence of toxic metabolites produced during the biodegradation process, which also need to be removed from the treated system in order to achieve a really clean environment.

### 3.4. Genome Annotation and Degradation Pathway Analysis

The number of metabolites identified throughout the IBP biodegradation process by *L. neptuniae* CSW11 (1-OH-IBP, 2-OH-IBP and CBX-IBP) was quite limited to propose a complete metabolic pathway for IBP degradation. As previously mentioned, the presence of some of these metabolites has been detected in various studies and different biodegradation pathways have been proposed for phylogenetically different microorganisms [26]. For this reason, the degradation pathway of IBP by *L. neptuniae* CSW11 was studied through an analysis of its genome to determine the potential genes involved and potential proteins related to IBP degradation comparing to the already mentioned published pathways. To achieve this objective, the whole-genome sequence of *Labrys neptuniae* CSW11 was annotated through the Rapid Annotation System Technology (RAST) server. The draft genome of this bacterial strain resulted in a total length of 7,596,229 bp with an average GC content of 63.8%, and the assembly yielded 84 contigs with an N50 of 146,164 bp. In order to determine potential genes involved in IBP degradation, a functional analysis of the genome was performed with the aid of the SEED annotation portal (Figure S3). A total of 90 genes related to the metabolisms of aromatic compounds were detected and classified into three different subcategories: metabolism of central aromatic intermediates (69 genes), metabolism of aromatic compounds (13 genes) and peripheral pathways for catabolism of aromatic compounds (eight genes).

As previously mentioned, there are different pathways for the degradation of IBP [26,71]. The first pathway for IBP biodegradation was proposed by Chen and Rosazza [72] based on the identification of two metabolites, ibuprofenol and ibuprofen acetate. Murdoch and Hay [20,21] reported that *Sphingomonas* sp. Ibu-2 was able to remove IBP and other similar compounds, and they identified a five-gene cluster *ipfABDEF* encoding enzymes involved in the biodegradation pathway. This cluster was not detected in the genome of *L. neptunieae* CSW11, but a certain percentage of identity with some phenylacetic acid catabolic enzymes was observed in the genome annotation of the strain CSW11 (Table S1). Also, Murdoch and Hay [73] reported IBP biotransformation by *Variovorax* sp. Ibu-1 to tri-OH-IBP by a ring meta-cleavage mechanism.

Lu et al. [40] used *Pseudoxanthomonas* sp.DIN-3 to degrade IBP. 1-OH-IBP and CBX-IBP were the first metabolites detected. Subsequent hydroxylations and oxidations led to the generation of various metabolites, even one with six hydroxyl groups.

Furthermore, Salgado et al. [29] reported the biodegradation of IBP by *Patulibacter medicamentivorans* proposing two main pathways. One of them involves the oxidation of the IBP molecule with the introduction of hydroxyl groups, and they observed the metabolites 1-OH-IBP and CBX-IBP, and proposed the enzyme Enoyl-CoA hydratase as responsible for such oxidation, which catalyzes β-oxidation by adding a proton and hydroxyl groups to an unsaturated β-carbon of the molecule. According to Salgado et al. [29], this enzyme is then converted into acyl-CoA and acetyl-CoA, generating energy in the form of NADH. In the present study, the metabolites 1- and 2-OH-IBP and CBX-IBP were also detected in the culture medium of *L. neptuniae* CSW11 during biodegradation assays.

Almeida et al. [39] carried out the first quantitative proteomic analysis combined with genetic information on the ibuprofen-degrading microorganism *Patulibacter* sp. strain I11 to investigate its degradation pathway. They proposed candidate enzymes potentially involved in the degradation pathways of IBP, including enoyl-CoA hydratase and acyl-CoA synthetase. Genes encoding the same enzymes enoyl-CoA hydratase and acyl-CoA synthetase were also identified in the genome of *L. neptuniae* CSW11 (Table 4).

On the other hand, Marchlewicz et al. [16,38] proposed a biodegradation pathway for *Bacillus thuringiensis* B1 where IBP is hydroxylated by an aliphatic monooxygenase, generating the first intermediate 2-OH-IBP, which is subsequently metabolized to 2-(4-hydroxyphenyl)-propionic acid and to 1,4-hydroquinone. The activity of the enzymes acyl-CoA synthase, hydroquinone monooxygenase and hydroxyquinol 1,2-dioxygenase, as well as the intermediates detected, confirmed this IBP degradation pathway. None of the enzymes described by Marchlewicz et al. [16] were detected, and therefore, *L. neptuniae* CSW11 could not degrade the intermediate 2-(4-hydroxyphenyl-) propionic acid by the same biodegradation pathway as that of *B. thuringiensis* B1. However, all genes encoding enzymes Hpa involved in the complete catabolism of a similar compound, 4-hydroxyphenylacetic acid [73], were identified in *L. neptuniae* CSW11 (Table 4).

**Figure 6:**
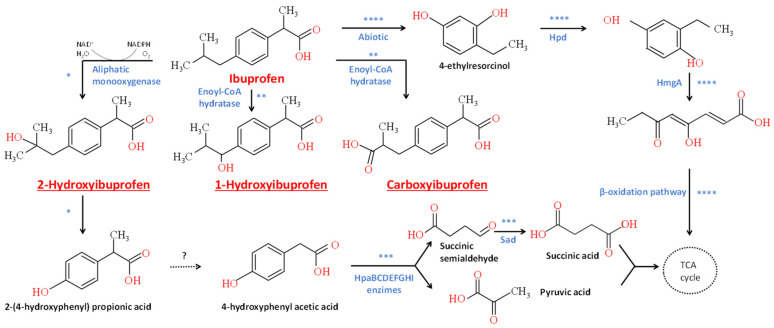
Potential biodegradation pathway of ibuprofen based on metabolites detected in the culture medium of *L. neptuniae* CSW11 during biodegradation assays (underlined) and catabolic enzymes described by Marchlewicz et al. * [16], Salgado et al. ** [29], Diaz et al. *** [73] and Li et al. **** [7], and identified in the genome annotation of the bacterial strain CSW11. Hpd (4-hydroxyphenylpyruvate dioxygenase), HmgA (homogentisate 1,2-dioxygenase), HpaBC (4-hydroxyphenyl acetate monooxygenase), HpaD (3,4 dihydroxyphenylacetate 2,3-dioxygenase), HpaE (5-carboxymethyl-2-hydroxymuconato semialdehyde dehydrogenase), HpaF (5-carboxymethyl-2-hydroxymuconato isomerase), HpaG (5-oxo-pent-3-ene-1,2,5-tricarboxylic acid), HpaH (2-oxo-hepta-3-ene-1,7-dioic acid hydratase), HpaI (2,4-dihydroxyhept-2-ene-1,7-dioic acid aldolase), Sad (succinic semialdehyde dehydrogenase).

Recently, Li et al. [7] used the marine bacteria *Pseudoalteromonas* sp. for IBP degradation and described, for the first time, a novel enzymatic–nonenzymatic coupling degradation mechanism. The initial stage of IBP transformation occurs by extracellular reactive oxygen species forming 4-ethylresorcinol through hydrogenation, isobutyl moiety cleavage, oxidation and decarboxylation. Then, this metabolite is further degraded by the intracellular enzymes 4-hydroxy-phenylpyruvate dioxygenase, homogentisate 1,2-dioxygenase, long-chain acyl-CoA synthetase, acetyl-CoA acyltransferase and enoyl-CoA hydratase. Genes encoding these enzymes were also detected in the genome of *L. neptunieae* CSW11 (Table 4); therefore, if this initial abiotic transformation of IBP occurs, the bacterial strain CSW11 has the necessary enzyme mechanism for the biodegradation of 4-ethylresorcinol. According to the genome analysis of the *L. neptuniae* CSW11 and the enzymes which have previously been reported as being potentially involved in the biotransformation of IBP, its metabolites or similar compounds, possible biodegradation pathways have been proposed (Figure 6).

## 4. Conclusions

Biotransformation of IBP using the bacterial strain *L. neptuniae* CSW11 isolated from sewage sludge from a WWTP has been carried out. It is important to highlight that the species *L. neptuniae* has not been previously described as a degrader of either IBP or any other organic contaminant. CSW11 reached an IC_50_ value of 217 mg L^−1^ IBP, being quite effective at low (1 mg L^−1^) and high (100 mg L^−1^) IBP concentrations, with increased removal as the glucose concentration increased. When using IBP 10 mg L^−1^, CSW11 in the presence of glucose 3 g L^−1^ showed 100% IBP removal in 7 days and a value of DT_50_ of just over a day. In the case of using IBP 100 mg L^−1^, DT_50_ was reduced from more than 30,000 days using 1 g L^−1^ glucose to only 29 days with 3 g L^−1^.

Three metabolites were identified throughout the IBP biotransformation process by *L. neptuniae* CSW11, 1-hydroxyibuprofen (1-OH-IBP), 2-hydroxyibuprofen (2-OH-IBP), and carboxyibuprofen (CBX-IBP), whose concentration declined drastically in the presence of glucose, indicating the convenience of using this growth substrate to both remove IBP and avoid the accumulation of its metabolites. In spite of that, ecotoxicity studies indicated that, even when IBP was completely removed by *L. neptuniae* CSW11, some of its toxic metabolites remained in solution, maintaining a certain degree of toxicity in the system.

The analysis of the *L. neptuniae* CSW11 genome was carried out and compared to genes of other IBP-degrading bacteria obtained from the literature, and various genes and enzymes that could be involved in IBP degradation by CSW11 were detected, proposing a potential IBP biodegradation pathway.

## Figures and Tables

**Table 1:** Optimized MRM parameters of the QqQ-MS determination of ibuprofen and its metabolites.

Compound	Retention Time (min)	Ion Polarity	Precursor Ion (*m*/*z*)	MRM 1 (Quantification)	MRM 2 (Confirmation)	CE (V)
Ibuprofen	8.80	Negative	205	205 > 205	205 > 161	0/4
1-hydroxyibuprofen	7.84	Positive	240	240 > 205	240 > 163	8/20
2-hydroxyibuprofen	7.64	Positive	240	240 > 205	240 > 107	12/36
Carboxyibuprofen	7.63	Positive	254	254 > 219	254 > 117	8/48

MRM: Multiple Reaction Monitoring; CE: collision energy.

**Table 2:** Kinetic parameters from ibuprofen biotransformation in solution after inoculation with *Labrys neptuniae* CSW11 at different initial concentrations of ibuprofen and glucose.

Initial Concentration of Ibuprofen (mg/L)	Initial Concentration of Glucose (g/L)	Kinetic Model	K_1_ (d^−1^)	K_2_ (d^−1^)	tb (d)	α (d^−1^)	β (d^−1^)	DT_50_ (d)	Extent of Removal (%)	R^2^	Err_scaled_	Calculated Χ^2^
1	1	FOMC	-	-	-	2.653	17.547	5.2	90.3	0.977	4.01	3.293
	3	HS	1.812	1.374	0.1	-	-	0.5	100.0	1.000	0.03	0.396
5	1	FOMC	-	-	-	0.599	3.623	7.9	73.8	0.991	1.94	0.861
	3	HS	0.526	5.711	2.6	-	-	1.3	100.0	1.000	0.0004	0.010
10	0	FOMC	-	-	-	0.233	3.462	64.5	40.0	0.971	1.99	0.568
	1	FOMC	-	-	-	2.906	23.561	6.3	81.7	0.944	6.63	3.102
	3	SFO	0.501	-	-	-	-	1.4	100.0	0.983	4.04	11.428
50	1	FOMC	-	-	-	0.307	4.699	40.2	36.3	0.855	5.48	3.566
	3	FOMC	-	-	-	0.154	0.036	3.2	61.5	0.993	1.49	0.513
100	1	FOMC	-	-	-	0.071	1.769	30,506	16.6	0.888	1.81	0.345
	3	FOMC	-	-	-	0.073	0.002	29.0	48.4	0.997	0.79	0.112

K_1_, K_2_: rate constants of biotransformation; tb: time at which K changes; DT_50_: time to reach half of IBP initial concentration; χ^2^ calculated values < χ^2^ corresponding tabulated value 12,592 (*p* < 0.05).

**Table 3:** Acute toxicity test towards *A. fischeri* of ibuprofen aqueous solutions at the beginning and at the end of the experiments (28 days) using *Labrys neptuniae* CSW11 in the presence of different initial ibuprofen and glucose concentrations.

	Time	Glucose (g L^−1^)	Ibuprofen Remaining Concentration (mg L^−1^)	TUs	Toxicity Level
*Labrys neptuniae* CSW11	0 days	0	10	2.25	Acute toxicity
	28 days	0	6.0	0.60	Slight acute toxicity
	28 days	1	1.0	0.87	Slight acute toxicity
	28 days	3	0	0.68	Slight acute toxicity
	0 days	0	50	3.98	Acute toxicity
	28 days	1	31.8	5.31	Acute toxicity
	28 days	3	19.2	5.80	Acute toxicity
	0 days	0	100	5.68	Acute toxicity
	28 days	1	83.4	8.72	Acute toxicity
	28 days	3	51.6	17.9	High acute toxicity

TUs: Toxic Units according to Persoone et al. [70].

**Table 4:** Enzymes potentially involved in the degradation of the ibuprofen metabolites 2-hidroxyibuprofen, 4-hydroxyphenyl acetic acid and 4-ethylresorcinol, annotated in the genome of *L. neptuniae* CSW11.

Metabolites	Enzyme Abbrev.	Contig	Peg Annotation	Putative Function
2-hydroxyibuprofen		15, 24	979, 2752	Aliphatic monooxygenase
1-hydroxyibuprofen		4, 12, 22, 30	428, 2614, 3521, 4653, 4727	Enoyl-CoA hydratase
Carboxyibuprofen				
4-hydroxyphenyl acetic acid	HpaA	43	5530	transcriptional regulator
	HpaB	43	5529	4-hydroxyphenylacetate 3-monooxygenase
	HpaC	43	5528	4-hydroxyphenylacetate 3-monooxygenase, reductase component
	HpaD	43	5507	3,4-dihydroxyphenylacetate 2,3-dioxygenase
	HpaE	43	5508	5-carboxymethyl-2-hydroxymuconate semialdehyde dehydrogenase
	HpaF	43	5509	5-carboxymethyl-2-hydroxymuconate delta-isomerase
	HpaG	43	5506	5-carboxymethyl-2-oxo-hex-3- ene-1,7-dioate decarboxylase
	HpaH	43	5505	2-oxo-hepta-3-ene-1,7-dioic acid hydratase
	HpaR	43	5531	Homoprotocatechuate degradative operon repressor
	Sad	4, 6, 27	3113, 3114, 4813, 6540	Succinate-semialdehyde dehydrogenase [NAD(P)+]
4-ethylresorcinol	Hpd	34	3886	4-hydroxyphenylpyruvate dioxygenase
	HmgA	12	504	Homogentisate 1,2-dioxygenase
		17	1651	Acyl-CoA synthetase
		5	5824	Acetyl-CoA acetyltransferase
		4, 12, 22, 30	428, 2614, 3521, 4653, 4727	Enoyl-CoA hydratase

## Data Availability

The original contributions presented in the study are included in the article/Supplementary Materials, further inquiries can be directed to the corresponding author.

## Supplementary Materials

The following supporting information can be downloaded at: https://www.mdpi.com/article/10.3390/jox15010005/s1, Table S1. Alignment of five-gene cluster ipfABDEF and the paa genes encoding enzymes involved in the biodegradation pathway of ibuprofen and other phenylacetic acids, respectively, which are annotated proteins in the genome of *L. neptuniae* CSW11; Figure S1. Graphical representation of the best fitting curve for *L. neptuniae* viability as a function of the logarithm of IBP concentration; Figure S2. LC-MS/MS chromatograms and mass spectra of measured ibuprofen and metabolites in the sample extract after seven days of degradation in the absence of glucose; Figure S3. SEED diagram showing whole genome annotation and functional gene analysis of *Labrys neptuniae* CSW11.
