# Supplementary material for: Removal of Ibuprofen in Water by Bioaugmentation with Labrys neptuniae CSW11 Isolated from Sewage Sludge—Assessment of Biodegradation Pathway Based on Metabolite Formation and Genomic Analysis
Source: J Xenobiot. 2024 Dec 31;15(1):5. doi: 10.3390/jox15010005 (PMC11755648; doi:10.3390/jox15010005)
Supplement: Supplementary file 1 [file jox-15-00005-s001.zip › jox-3215667-supplementary.pdf]

**Figure S1.** Graphical representation of the best fitting curve for *L. neptuniae* viability as a function of the logarithm of IBP concentration.

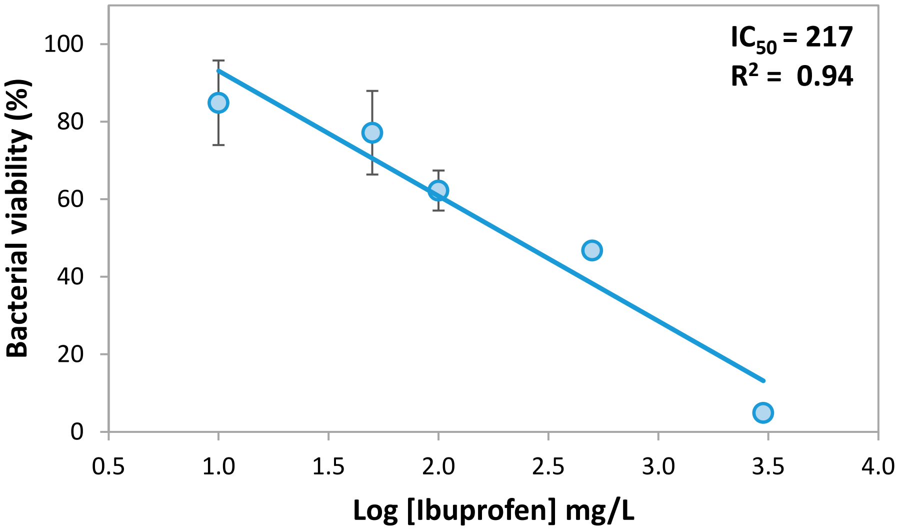

**Figure S2.** LC-MS/MS chromatograms and mass spectra of measured ibuprofen and metabolites in the sample extract after seven days of degradation in the absence of glucose.

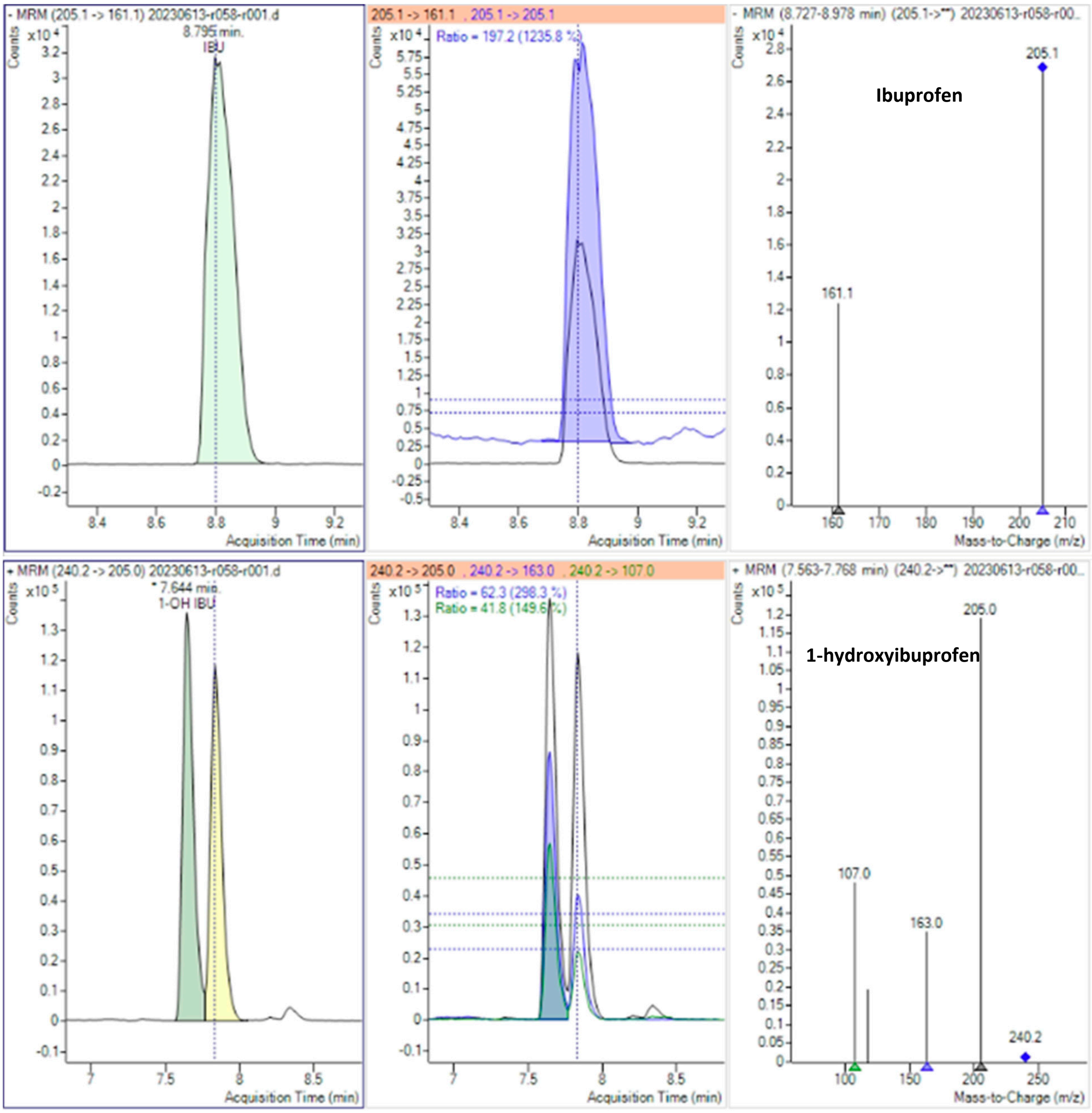

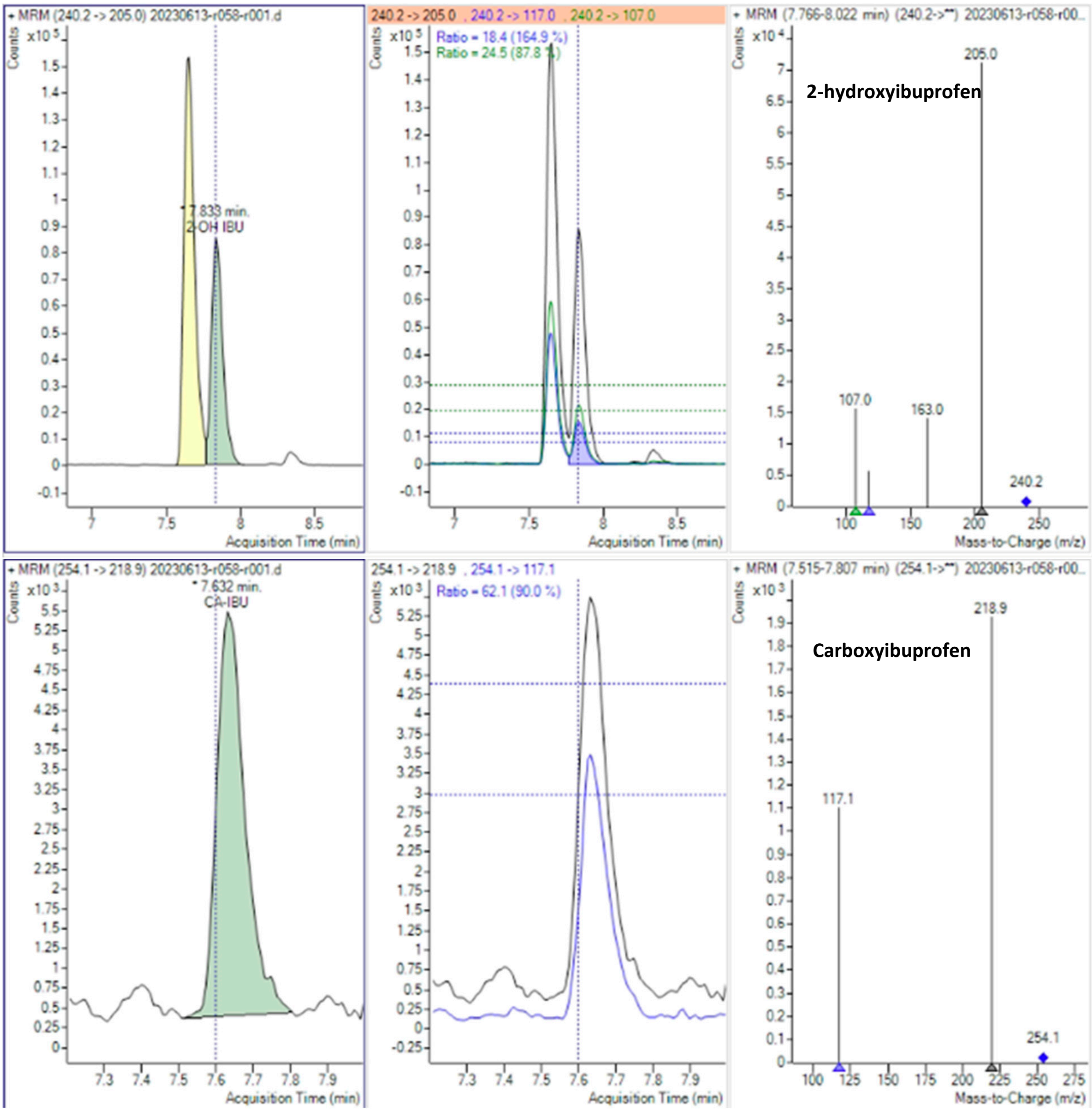
+ MRM (240.2 -> 205.0) 20230613-r058-r001.d
Counts
x10 5
7.833 min.
2-OH IBU
Acquisition Time (min)
240.2 -> 205.0 , 240.2 -> 117.0 , 240.2 -> 107.0
Ratio = 18.4 (164.9 %)
Ratio = 24.5 (87.8 %)
+ MRM (7.766-8.022 min) (240.2->**) 20230613-r058-r00...
x10 4
2-hydroxyibuprofen
205.0
107.0
163.0
240.2
Mass-to-Charge (m/z)
+ MRM (254.1 -> 218.9) 20230613-r058-r001.d
x10 3
7.632 min.
CA-IBU
254.1 -> 218.9 , 254.1 -> 117.1
Ratio = 62.1 (90.0 %)
+ MRM (7.515-7.807 min) (254.1->**) 20230613-r058-r00...
Carboxyibuprofen
218.9
117.1
254.1

**Figure S3.** SEED diagram showing whole genome annotation and functional gene analysis of *Labrys neptuniae* CSW11.

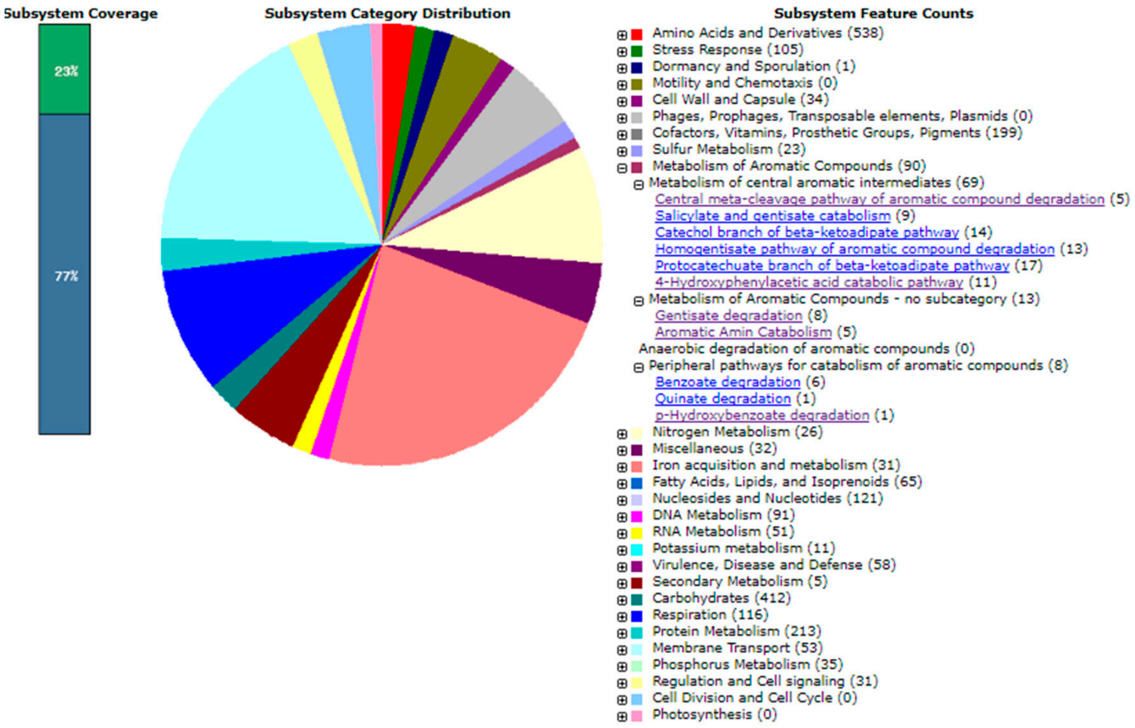

**Table S1.** Alignment of five-gene cluster *ipf*ABDEF and the *paa* genes encoding enzymes involved in the biodegradation pathway of ibuprofen and other phenylacetic acids, respectively, which are annotated proteins in the genome of *L. neptuniae* CSW11.

| Gen | Organism | Enzyme | E-value | Identity (%) | Putative function |
|---|---|---|---|---|---|
| *ipfA* | Sphingomonas sp. Ibu-2 | Ibuprofen CoA dioxygenase large subunit | 2E-49 | 30 | Benzoate 1,2-dioxygenase alpha subunit |
| *ipfB* | Sphingomonas sp. Ibu-2 | Ibuprofen-CoA dioxygenase small subunit | 7E-08 | 30 | Benzoate 1,2-dioxygenase beta subunit |
| *ipfD* | Sphingomonas sp. Ibu-2 | Thiolase/acyl transferase | 0.65 | 24 | 3-ketoacyl-CoA thiolase @ Acetyl-CoA acetyltransferase |
| *ipfE* | Sphingomonas sp. Ibu-2 | - | 0.66 | 39 | hypothetical protein |
| *ipfF* | Sphingomonas sp. Ibu-2 | Ibuprofen CoA ligase | 1E-52 | 31 | Long-chain-fatty-acid--CoA ligase |
| *paaA* | E. coli K-12 | - | - | - | - |
| *paaB* | E. coli K-12 | phenylacetyl-CoA 1,2-epoxidase, subunit B | 0.75 | 55 | Hypotetical protein |
| *paaC* | E. coli K-12 | phenylacetyl-CoA 1,2-epoxidase, structural subunit | 0.80 | 36 | aminoacid ABC transporter, ATP-binding protein |
| *paaE* | E. coli K-12 | phenylacetyl-CoA 1,2-epoxidase, reductase subunit | 2E-43 | 32 | Flavodoxin reductases (ferredoxin-NADPH reductases) family 1 |
| *paaF* | E. coli K-12 | putative 2,3-dehydroadipyl-CoA hydratase | 2E-71 | 46 | Enoyl-CoA hydratase |
| *paaG* | E. coli K-12 | putative ring 1,2-epoxyphenylacetyl-CoA isomerase | 2E-72 | 43 | Enoyl-CoA hydratase |

| | | | | | |
|---|---|---|---|---|---|
| *paaH* | E. coli K-12 | 3-hydroxyadipyl-CoA dehydrogenase | 1E-61 | 37 | 3-hydroxybutyryl-CoA dehydrogenase |
| *paaI* | E. coli K-12 | phenylacetyl-CoA thioesterase | 6E-04 | 30 | cytosolic long-chain acyl-CoA thioester hydrolase family protein |
| *paaJ* | E. coli K-12 | beta-ketoadipyl-CoA thiolase | 1E-174 | 65 | Acetyl-CoA acetyltransferase / 3-oxoadipyl-CoA thiolase |
| *paaK* | E. coli K-12 | phenylacetate-CoA ligase | 0.001 | 25 | 2,3-dihydroxybenzoate-AMP ligase of siderophore biosynthesis |
| *paaL* | E. coli K-12 | - | - | - | - |
| *paaM* | E. coli K-12 | - | - | - | - |
| *paaX* | E. coli K-12 | DNA-binding transcriptional repressor PaaX | 0.13 | 26 | Dipeptide-binding ABC transporter, periplasmic substrate-binding component |
| *paaY* | E. coli K-12 | 2-hydroxycyclohepta-1,4,6-triene-1-carboxyl-CoA thioesterase | 5E-38 | 39 | Carbonic anhydrase-like protein MJ0304 |
| *paaZ* | E. coli K-12 | fused 3-oxo-5,6-dehydrosuberyl-CoA semialdehyde dehydrogenase and oxepin-CoA hydrolase | 2E-22 | 25 | Succinate-semialdehyde dehydrogenase [NAD(P)+] |
